# Participation of paediatric patients in primary dental care before and after a dental general anaesthetic

**DOI:** 10.1007/s40368-021-00624-3

**Published:** 2021-06-04

**Authors:** J. F. Large, A. J. Keightley, A. Busuttil-Naudi

**Affiliations:** Paediatric Dentistry Department, Edinburgh Dental Institute, Lauriston Building, Lauriston Place, Edinburgh, EH3 9HA Scotland, UK

**Keywords:** Dental general anaesthetic (DGA), Child, Caries, Participation, Attendance, Prevention

## Abstract

**Purpose:**

The aim of this retrospective study is to determine children’s attendance and experience of preventative interventions and operative treatment (restorations and extractions) with their primary care dentist (PCD) in the 12 months before and after their caries management under dental general anaesthetic (DGA).

**Methods:**

A record of all children who had an elective DGA in 2016 across two hospital sites was retrospectively obtained (*n* = 1308). A representative sample of 300 was randomly selected encompassing 114 dental practices. An online questionnaire to the children’s PCDs collated quantitative and qualitative data regarding participation in the pre- and post-DGA period.

**Results:**

Data was collated and analysed for 80 children (mean age: 6 years 10 months [SD = 2.49; range: 2 years 1 month – 14 years 3 months]; equal sex distribution) with 43 responding PCDs. Attendance for examination declined significantly from 85% (*n* = 68) pre-DGA to 57.5% (*n* = 46) post-DGA (*p* ≤ 0.001). Attendance at emergency appointments pre-DGA was high (33.75% [*n* = 27]); a significant reduction post-DGA was recorded (*p* ≤ 0.001). Over one third of children (37.5% [*n* = 30]) did not receive any form of preventative intervention over 24 months. A non-significant reduction in the provision of operative treatment was observed post-DGA (*p* = 0.06 [fill, primary];* p* = 0.78 [fill, permanent];* p* = 0.66 [ext, primary]). No statistical difference between age and treatment experience was found. Qualitative analysis revealed challenges in providing care included behavioural difficulties and poor attendance.

**Conclusion:**

Improvements are required in strategies employed to support high caries risk children pre- and post-DGA to facilitate a higher incidence of attendance and preventative intervention with PCDs.

## Introduction

A continual challenge within paediatric dentistry is facilitating the reduction in caries experience and caries inequality in children. It is well documented that children with dental general anaesthetic (DGA) experience for caries management are a high caries risk group (Foster et al. 2006; Savanheimo and Vehkalahti 2014; EzEldeen et al. 2015; Haworth et al. 2017; Tilja et al. 2019). Caries risk is also increased when children are from poorer socio-economic backgrounds, from certain ethnic groups (Ramdaw, Hosey and Bernabe 2017) and when they are under the age of 4 years at the time of their first DGA (Kakaounaki et al. 2006; Lawson et al. 2017; Grindefjord et al. 2018).

The functional, social and psychological impacts on this cohort of children are also well reported. Commonly reported factors include pain, school absence, disturbed sleep, difficulty eating, emotional upset as well as wider family impacts such as parental time-off work and financial implications (Thomson and Malden 2011; Finucane 2012; Yawary et al. 2016; de Souza et al. 2017).

There is a relatively sparser body of literature assessing the engagement of children who require a DGA with their primary care dentist (PCD). Irregular dental attendance amongst this high caries risk cohort is reported both before and following treatment with children largely attending the dentist only when in pain with regular recall documented as low as 58% (EzEldeen et al. 2015; Goodwin et al. 2015a, b). Infrequent attendance limits opportunities for the delivery of prevention which is recognised as key to help reduce caries and minimise the need for further interventional dental treatment (Savanheimo et al. 2012; Tilja et al. 2019).

Locally, in keeping with normal practice in the United Kingdom (UK), the patient journey usually starts with a referral from their PCD for caries management under DGA. A full assessment is conducted by a member of the paediatric dentistry team and should a DGA be deemed appropriate the patient is placed on the DGA waiting list. A general anaesthetic is frequently recommended to facilitate caries management for children, either due to limited cooperation in the very young, or severity of caries or anxiety and medical co-morbidities, or a combination of any of these (Savanheimo et al. 2012; Savanheimo and Vehkalahti 2014; Lawson et al. 2017).

All DGAs in the UK are carried out in a hospital setting. Locally DGAs are carried out at two sites, either the local children’s hospital or a local district general hospital. On completion of the DGA, a hospital discharge letter is issued to the PCD detailing treatment undertaken and requesting follow-up care.

The aim of this retrospective study was to determine children’s attendance and experience of preventative interventions and operative treatment with their PCD in the 12 months before and after their caries management under DGA. The null hypothesis is there is no difference in the participation of paediatric patients pre- and post-DGA with their PCDs.

## Materials and methods

This retrospective study received ethical approval from the Proportionate Review Sub-Committee, Office for Research Ethics Committees Northern Ireland (ORECNI). REC Reference: 19/NI/0177. The study was based on a questionnaire study design collating quantitative and qualitative data from children’s PCDs.

### Data collection and participants

A record of all children who had an elective DGA from 1 January 2016 to 31 December 2016 was retrospectively obtained from a theatre booking and scheduling database (Operating Room Scheduling and Office System [ORSOS]). All children aged ≤ 16 years at the time of their DGA were included. The two hospital settings that provide paediatric DGA were included in the study; one a district general hospital and the other a specialist children’s hospital. Each child’s DGA had been treatment planned directly, or under the supervision of, a consultant or specialist in paediatric dentistry following referral into the paediatric dental service from their PCD. Children who received treatment on emergency theatre lists were not included.

The number of elective DGAs in 2016 was 1308 including caries and non-caries management. A representative sample of 10% was chosen. In order to compensate for a potential partial response rate from dental practices, a pragmatic decision was made to obtain a random sample of 300 children for inclusion in the study.

The sample was obtained by assigning each child a unique identifier number (1–1308) and utilising a random number generator to identify 300 unique children.

**Fig. 1:**
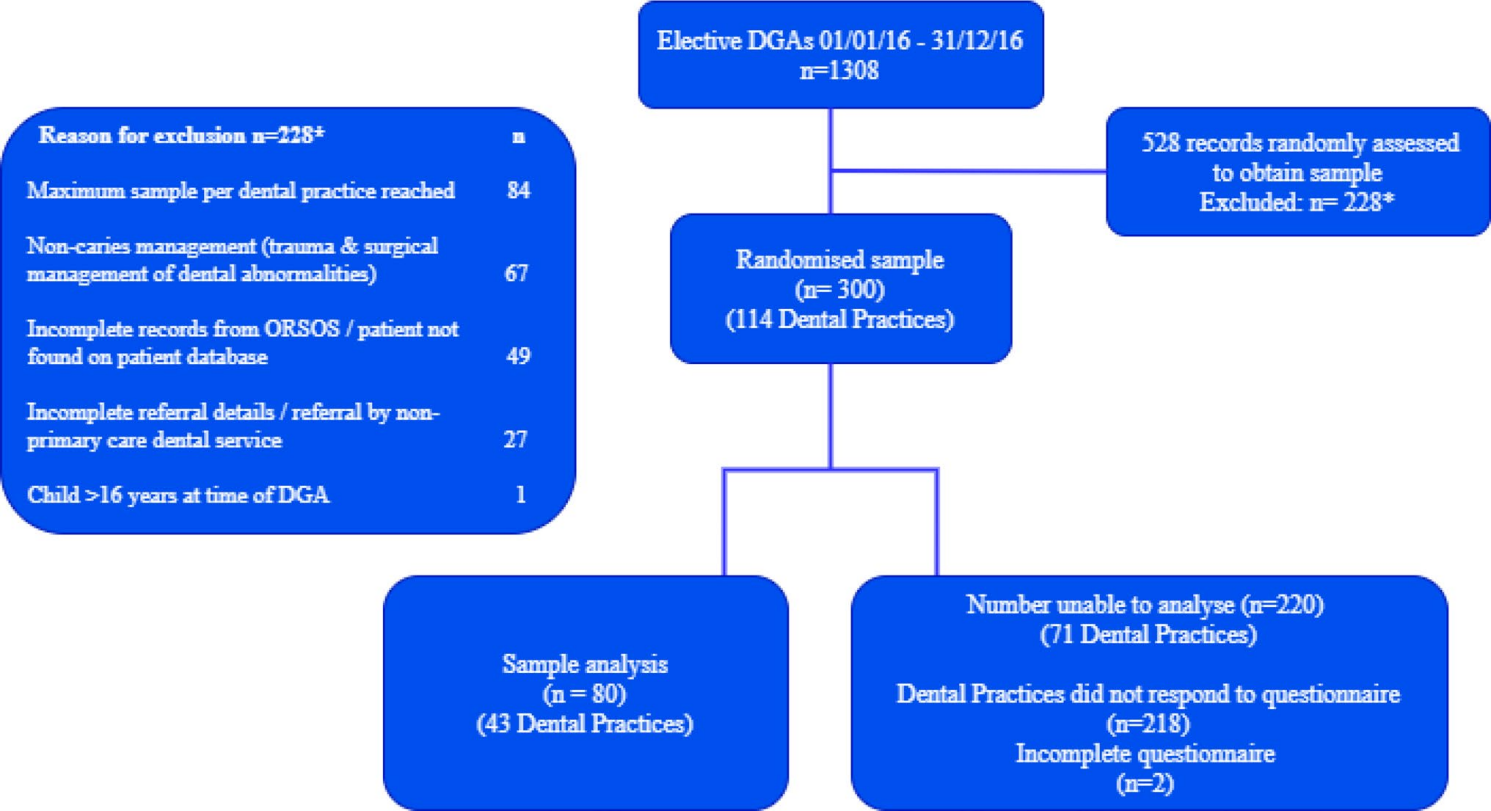
A flow diagram of the study

The dental records of selected children were viewed to obtain further information including name, date of birth, date of DGA(s), reason for treatment and name and address of their referring PCD. In instances when a complete data set was irretrievable, such as lack of documentation of the PCD, the child was excluded and a further child randomly recruited. Similarly, if a child’s DGA was for treatment other than caries management (i.e. trauma or surgical management of dental abnormalities), they were excluded and a further child randomly selected (Fig. 1).

The selected children were grouped together by their referring primary care dental practices and a maximum of five children per dental practice was included to restrict the study burden on practices and to minimise the study impact should a practice elect not to participate in the study. Further random selection of children was performed until a full sample of 300 children were identified.

An online questionnaire was developed through Joint Information Systems Committee (Jisc); an online survey tool designed for academic research, education and public sector organisations. Quantative data were obtained from two multiple choice answer questions concerning each child’s attendance and treatment experiences in the 12 months pre- and post-DGA. Participants (dentist or any other member of the dental team) were asked to indicate if the child had experienced any of the following in the 12 months prior to the DGA:

- Dental examination (exam);
- oral hygiene instruction and / or diet advice (OHI / Diet advice);
- fluoride varnish (FV);
- fissure sealant(s) (FS);
- restoration(s) of primary teeth including preformed metal crowns (fill [primary]);
- restoration(s) of permanent teeth (fill [permanent]);
- extraction(s) of primary teeth (ext [primary]);
- extraction(s) of permanent teeth (ext [permanent]);
- emergency appointment for pain and / or infection (emergency).

The same answer options were available concerning the 12 months post-DGA alongside the option to indicate if the child attended for post-operative review as requested in the child’s hospital discharge letter. The survey concluded with a free text box for the participant to enter any supporting comments of benefit to the study.

Practices were sent an invitation letter to participate in the study including the necessary patient-identifiable information and instructions on accessing and completing the online questionnaire. After 6 weeks, reminder letters were sent to non-responding practices to encourage participation. A final letter containing the original invitation letter, alongside a reminder letter, was sent after a further four weeks as required. Data collection continued for another 4 weeks preceding closure of the online questionnaire.

Throughout data collection and analysis, measures were taken to ensure data were kept secure. Letters containing patient identifiable information were sent by recorded delivery or between secure National Health Service (NHS) email accounts and only anonymous data were returned via the online questionnaire. The online security of Jisc was investigated and approved prior to its use in the study.

### Data analysis

Simple descriptive analyses were undertaken to report the mean age (SD, range) of the sample.

Statistical analysis was performed with the ‘Statistical calculator’ by SciStat. McNemar’s test (with Yates’s correction of 0.5) was performed to determine any statistically significant differences between pre-DGA and post-DGA data. A significance level was set at 5% (*p* ≤ 0.05).

Statistical analysis was also performed with IBM SPSS Statistics v.24 with Pearson chi-square tests to determine potential significant differences between treatment experience and age. A significance level was set at 5% (*p* ≤ 0.05).

## Results

### Response rate

Overall, 43 of 114 (37.7%) dental practices responded to the online questionnaire which included 82 of 300 children (27.3%). Following exclusion of two incomplete returned questionnaires, a total of 80 children (26.7%) were included in the analysis.

### Participants

**Fig. 2:**
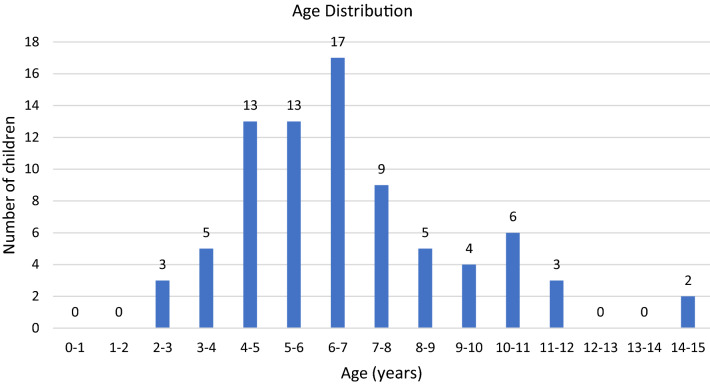
Age distribution of the sample

The 80 participants had a mean age of 6 years 10 months (SD = 2.49; range 2 years 1 month – 14 years 3 months) and included an equal proportion of males (*n* = 40; 50%) and females (*n* = 40; 50%). Figure 2 shows over half of the sample were aged between 4 and 7 years (*n* = 43; 54%) Very few children in the sample were younger than 3 years or older than 12 years (*n* = 5). Children were stratified by age into the following categories 0–4 years (*n* = 21), 5–9 years (*n* = 48), 10–16 years (*n* = 11).

### Examination

**Fig. 3:**
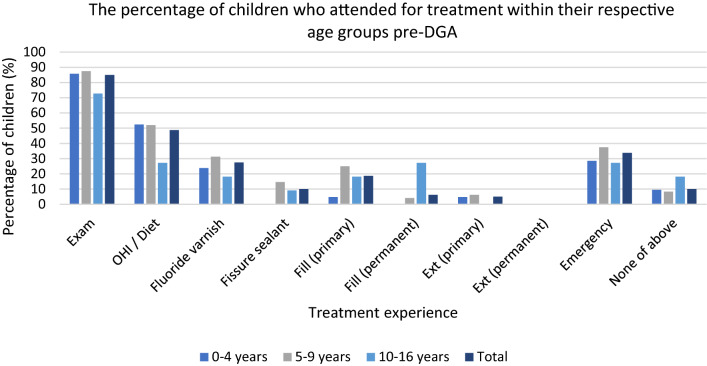
Treatment and attendance 12 months pre-DGA

It was reported that 85% (*n* = 68) of all children attended for an examination within the 12 months prior to their DGA (Table 1). Figure 3 illustrates how children aged ≤ 9 years made up a higher proportion of those attending for an examination with 86% (*n* = 18) of children aged 0–4 years attending and 88% (*n* = 42) children 5–9 years. The lowest rate of attendance for examination was reported in the 10–16 years age group (73% [*n* = 8]).

**Table 1:** Treatment and attendance 12 months pre-DGA reported by PCDs

	Number of children per age *n* (%)			
	0–4 years (*n* = 21)	5–9 years (*n* = 48)	10–16 years (*n* = 11)	Total (*n* = 80)
T*reatment recorded*				
Exam	18 (85.7%)	42 (87.5%)	8 (72.7%)	68 (85%)
OHI/Diet advice	11 (52.4%)	25 (52.1%)	3 (27.3%)	39 (48.8%)
FV	5 (23.8%)	15 (31.3%)	2 (18.2%)	22 (27.5%)
FS	0 (0%)	7 (14.6%)	1 (9.1%)	8 (10%)
Fill (primary)	1 (4.8%)	12 (25%)	2 (18.2%)	15 (18.8%)
Fill (permanent)	0 (0%)	2 (4.2%)	3 (27.3%)	5 (6.3%)
Ext (primary)	1 (4.8%)	3 (6.3%)	0 (0%)	4 (5%)
Ext (permanent)	N/A	0 (0%)	0 (0%)	0 (0%)
Emergency appointment	6 (28.6%)	18 (37.5%)	3 (27.3%)	27 (33.8%)
None of the above (no reported contact with PCD)	2 (9.5%)	4 (8.3%)	2 (18.2%)	8 (10%)

OHI = Oral hygiene instruction; FV = Fluoride varnish; FS = Fissure sealant; Fill = restoration(s) of primary teeth including preformed metal crowns/restoration of permanent teeth; Ext = extraction(s) of primary teeth/permanent teeth; N/A = Not applicable

**Fig. 4:**
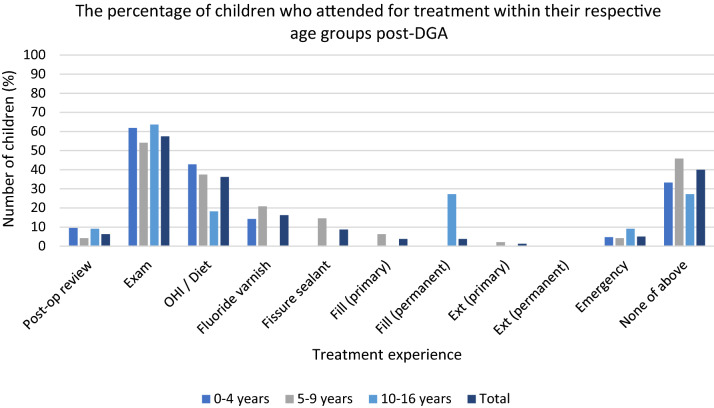
Treatment and attendance 12 months post-DGA

The proportion of all children reported to have attended an examination within 12 months post-DGA is significantly lower at 57.5% (*n* = 46; p =  < 0.001 [with Yates’s correction], 95% confidence interval [1.27–2.43]) with only 6.3% (*n* = 5) attending for post-operative review (Table 2). Figure 4 illustrates a different pattern to that seen pre-DGA, with children aged 10–16 years having the highest attendance for examination post-DGA (64% [*n* = 7]).

**Table 2:** Treatment and attendance 12 months post-DGA reported by PCDs

	Number of children per age * n* (%)			
	0–4 years (*n* = 21)	5–9 years (*n* = 48)	10–16 years (*n* = 11)	Total (*n* = 80)
*Treatment recorded*				
Post-operative review	2 (9.5%)	2 (4.2%)	1 (9.1%)	5 (6.3%)
Exam	13 (61.9%)	26 (54.2%)	7 (63.6%)	46 (57.5%)
OHI/Diet advice	9 (42.9%)	18 (37.5%)	2 (18.2%)	29 (36.3%)
FV	3 (14.3%)	10 (20.8%)	0 (0%)	13 (16.3%)
FS	0 (0%)	7 (14.6%)	0 (0%)	7 (8.8%)
Fill (primary)	0 (0%)	3 (6.3%)	0 (0%)	3 (3.8%)
Fill (permanent)	0 (0%)	0 (0%)	3 (27.3%)	3 (3.8%)
Ext (primary)	0 (0%)	1 (2.1%)	0 (0%)	1 (1.3%)
Ext (permanent)	N/A	0 (0%)	0 (0%)	0 (0%)
Emergency appointment	1 (4.8%)	2 (4.2%)	1 (9.1%)	4 (5%)
None of the above (no reported contact with PCD)	7 (33.3%)	22 (45.8%)	3 (27.3%)	32 (40%)

OHI = Oral hygiene instruction; FV = Fluoride varnish; FS = Fissure sealant; Fill = restoration(s) of primary teeth including preformed metal crowns/restoration of permanent teeth; Ext = extraction(s) of primary teeth/permanent teeth; N/A = Not applicable

### Preventative interventions

Tables 1 and 2 show an overall low reporting of preventative interventions (OHI / Diet advice, FV and FS) both pre- and post-DGA. The provision of OHI / Diet advice was reported more frequently than FV or FS placement. The provision of these interventions showed a decline post-DGA but this was not statistically significant (*p* = 0.12; *p* = 0.17; *p* = 0.91 [with Yates’s correction]).

Children aged 10–16 years appear to have received the least overall preventative interventions with the exception of FS pre-DGA. The majority of preventative intervention was received by children aged 5–9 years (Table 1 and 2). However, comparison of pre-DGA and post-DGA age groups revealed no statistically significant differences with regards to OHI / Diet advice (chi^2^ = 5.36, *P* = 0.253, 4 df) and FV (chi^2^ = 3.71, *P* = 0.447, 4 df). Comparison of FS experience amongst children aged 5–9 years and 10–16 years was also non-significant (chi^2^ = 1.60, *P* = 0.206, 1 df).

Further analysis revealed 30 children (37.5%) did not receive any form of preventative intervention. There was no significant difference in age between children who did not receive any preventative intervention and those who received at least one intervention (*p* = 0.51, *t* test).

### Operative treatment

Table 1 shows that 18.8% (*n* = 15) of all children had experience of a restoration(s) in primary teeth and 6.3% (*n* = 5) in permanent teeth pre-DGA. The mean age of a child experiencing restoration of a primary tooth was 7 years 3 months (SD = 1.6) and 10 years 3 months (SD = 2.31) for restoration of a permanent tooth.

Restorations in primary teeth pre-DGA were more frequently placed in children aged 5–14 years with very few placed in children ≤ 4 years. Children aged 10–16 years had the greatest experience of restorations in permanent teeth pre-DGA (27% [*n* = 3]).

At 12 months post-DGA there was a lower overall proportion of all children who had restorations placed in primary and permanent teeth compared to pre-DGA levels (*p* = 0.06; *p* = 0.78 [with Yates’s correction]). However, the same incidence of restorations in permanent teeth pre-DGA and post-DGA amongst children aged 10–16 years was reported (27.3% [*n* = 3]) (Tables 1 and 2). No statistically significant differences were found between age and experience of restorations in primary teeth (chi^2^ = 4.58, *P* = 0.101, 2 df). A significant difference was determined between age and experience of restorations in permanent teeth (chi^2^ = 10.16, *P* = 0.001, 1 df).

The lowest prevalence across all treatment experience was extraction. No extractions of permanent teeth in the 12 months pre- or post-DGA were recorded. The decrease in pre-DGA to post-DGA primary extractions was not statistically significant (*p* = 0.66 [with Yates’s correction]). There was no significant difference between age and extraction of a primary tooth (chi^2^ = 1.17, *P* = 0.558, 2 df).

Overall, there was no significant association between experience of pre-DGA preventative intervention(s) and experience of operative treatment post-DGA or pre- and post-DGA (*p* > 0.05).

### Emergency care

One-third of all children attended an emergency appointment for dental pain and/or infection in the 12 months pre-DGA (33.75% [*n* = 27]) (Table 1). Figure 3 illustrates the highest proportion of children attending by age category was those aged 5–9 years (36% [*n* = 18]).

Post-DGA a much smaller proportion of all children attended (5% [*n* = 4]) (Table 2 and Fig. 4). The reduction in attendance post-DGA was statistically significant (*p* =  < 0.001 [with Yates’s correction] 95% confidence interval [1.31 to 2.50]).

### ‘None of the above’/Reports of no assessment or treatment

Tables 1 and 2 show 10% of all children were reported to not have had any pre-DGA treatment or assessment experience by their PCD with this increasing to 40% post-DGA. Qualitative analysis provides insight into this apparent lack of attendance (Table 3). Common themes emerging include a complete or delayed lack of engagement with their PCD post-DGA and transfer of care to another dental practice or specialist dental service.

**Table 3:** Challenges reported from PCDs in providing care to high caries risk children

Challenge	Example(s)	*N*
Behavioural e.g. learning difficulties/lack of cooperation	“Patient has autism and co-operation very poor.” “Very anxious little boy, adopted, this was his third 'mum' [and he] carried a lot of worry and was very uncooperative due to his past.”	9
Medical condition	“Patient has [a] complex medical history including growth hormone [treatment] and high calorie diet.”	1
Poor attendance/lack of engagement	“Patient has not attended since referral in 2015.” “Referred for Childsmile but appointments missed or cancelled.” “Attended pre-DGA with pain and infection. Patient has been brought only once following DGA.”	26
Family live abroad and access dental care when in the United Kingdom	“The patient’s family are expats and live in Africa. They access dental treatment only when they are back in the UK”	1
Family wishes	“Patient’s mum refused to consent to second DGA appointment and direct restorations were attempted but not successful in March due to poor cooperation.”	1
Lack of continual care (change in primary dental care practice provider)	“Patient was new to the practice in 2015 and never returned. Health board informed us that they have transferred to another practice.”	5

### Repeat DGA

The repeat DGA rate in the study sample analysed was 0.8% (*n* = 1) involving a child with dental caries and a diagnosis of autism aged 10 years 7 months at the time of their first DGA and 12 years at the time of their second DGA. On analysis of the full sample invited to participate (*n* = 300), the repeat DGA rate was 1% (*n* = 3).

## Discussion

This research clearly shows that attendance for regular examination and preventative interventions in primary dental care for high caries risk children is suboptimal. Attendance declined significantly from 85% (*n* = 68) pre-DGA to 57.5% (*n* = 46) post-DGA. Similar low post-DGA recall of 58% has been reported in a study in north-west England suggesting a possible negative shift in attitude towards attendance post-DGA (Goodwin, Sanders and Pretty 2015a, b). The driver for this change is likely to be multifactorial, for example increased dental anxiety or a reduced perceived treatment need post-DGA. The prevalence of dental anxiety has been reported to be as much as 3.62 times greater amongst children who have experienced a DGA (Savanheimo and Vehkalahti 2014; Haworth et al. 2017). Such anxiety may be sufficient enough to lead to avoidance of dental attendance or contribute to difficulties in cooperation for those children that do attend dental appointments (Nuttall et al. 2008; Savanheimo and Vehkalahti 2014). One study reported over half of children demonstrated continued difficulties with cooperation in the dental environment (54%) and over half expressed dental fear (53%) at follow-up appointments (Savanheimo and Vehkalahti 2014). One method proposed to address this amongst young children is play therapy prior to and at the time of DGA (Schwartz Albino and Tedesco 1983).

In addition, significantly lower post-DGA attendance may be associated with differences in practice policies to encourage attendance, such as reminders, whilst management of children not brought (WNB) are likely to vary. Qualitative analysis revealed poor attendance and lack of engagement as the most common theme to emerge from PCDs regarding challenges to delivering dental care to this cohort.

Similarly, engagement with post-operative review was extremely low (6.3% [*n* = 5]) despite all hospital discharge letters requesting primary care to facilitate a review. Attendance reported in the literature is variable but considerably higher than reported in this study from 39–88%, 1–2 weeks post-DGA (Amin et al. 2015; Goodwin et al. 2015a, b). However, even with initial higher levels of engagement, a decline in subsequent attendance as low as 36% at 6 months post-DGA and 26–45% 3 years post-DGA is reported (Al-Malik and Al-Sarheed 2006; Amin et al. 2015). Lack of engagement at post-operative review may be a reflection of the perceived unimportance of this visit, failure of a practice to arrange a review or failure to engage from the family. Alternatively, a post-operative review may have taken place but recorded as an ‘exam’ by the PCD and, as such, perhaps overlooked when completing the questionnaire. Although this study confirmed the presence of the original discharge letter in the patient’s hospital notes, practices were not requested to confirm past receipt of the letter as part of the study. Receipt of the letter could be explored in future work to deduce if lack of receipt could be a contributory factor behind low attendance at post-operative review and highlight an area for service improvement.

One third of all children (33.8% [*n* = 27]) attended an emergency dental appointment with pain and / or infection in the 12 months pre-DGA. Other studies have demonstrated much greater attendance in cohorts ≤ 6 years of age (44%) and ≤ 5 years (88%) (Kvist et al. 2014; McAuliffe et al. 2017). Reassuringly, emergency attendance in this study significantly reduced post-DGA to just 5% (*n* = 4) suggesting comprehensive management of caries and associated pain and infection at the time of DGA. However, 5% may not be a true reflection of requirement for emergency dental care with access to emergency care via alternative pathways possible, a reported high lack of attendance post-DGA (40% [*n* = 32]) and with potentially significant barriers such as dental anxiety. A study in Belgium reviewed children twelve years post-DGA reporting that, despite 86% agreeing biannual dental visits would be good practice, 24% would only attend when in pain (EzEldeen et al. 2015). Also, qualitative analysis revealed some children had changed primary dental care practice following their DGA (*n* = 5) and reports of their ongoing dental care including any emergency appointments was outwith the scope of this study.

The provision of OHI / Diet advice, FV and FS appear inadequately low considering the recommendation for enhanced prevention for all high caries risk children (Public Health England 2017; SDCEP 2018). Delivery of preventative advice was the most commonly utilised form of prevention with 48.8% (*n* = 39) children receiving this pre-DGA. A retrospective cohort study in Finland found that, similarly, only 52% children had received preventative advice prior to referral to specialist care (Savanheimo et al. 2012). These shortfalls may be explained by difficult behaviour management, 9 PCDs acknowledged this as a challenge (Table 3), or reflect possible deficiencies in the national remuneration system. Mandatory attendance at pre-DGA prevention clinics has been proposed to increase opportunities to deliver prevention and encourage participation (Goodwin et al. 2015a, b). However, the greater utilisation of advice over other preventative interventions, although important and perhaps reflective of difficult behaviour management, is inconsistent with research that improved knowledge does not always translate into behaviour change (Amin and Harrison 2006; Jankauskiene et al. 2017). A further study reported only 47.7% of parents positively made changes to their child’s diet following advice and 17.7% did not change diet nor oral hygiene practices (Valera et al. 2016).

Motivational interviewing is an alternative method of delivering prevention with a greater potential for positive dental health behaviour changes, alongside a structured prevention programme, than more traditional methods (Pine et al. 2015). Therefore, reorientation of dental undergraduate and postgraduate teaching on prevention, with a focus on motivational interviewing, may help with future efforts to drive the desired behaviour change amongst children and their families.

Enhanced prevention remains best practice (Public Health England 2017; SDCEP 2018). However, it is unknown which method(s) of prevention delivery are the most effective, whether this includes pre-DGA prevention clinics or motivational interviewing. Ideally, future research is required to identify the most effective, efficient and cost-effective way of delivering prevention.

Moreover, the overall incidence of restorations in permanent teeth by PCDs pre-DGA appears low (6.3% [*n* = 5]) but should be interpreted with the understanding that 26.3% of the sample (*n* = 21) were < 5 years. Also, the definition of a “restoration” was not explored and could indicate a small preventative resin restoration or an extensive multi-surface restoration.

A higher incidence of restoration of primary teeth by PCDs pre-DGA was reported (18.75% [*n* = 15]). Although not statistically significant, very few restorations were placed in children ≤ 4 years (5% [*n* = 1]) compared with older children aged 5–9 years (25% [*n* = 12]) who would still be expected to have retained most of their primary dentition. Similarly, a study in Lithuania of 144 children aged 2–6 years reported a low incidence of restored and extracted teeth with young age, dental anxiety and lack of cooperation outlined as possible explanatory factors (Jankauskiene et al. 2013).

Extraction was the least common intervention in primary care. Lack of cooperation, anxiety, young age and overall treatment need may have precluded a greater incidence of extractions by PCDs. However, despite the low levels of caries management reported post-DGA, with the exception of restoration of permanent teeth, there was still a requirement to treat new or residual caries (relapse). New carious lesions have been reported as soon as six months post-DGA in up to 37–52% of cases which may be an under-estimate of disease prevalence given suboptimal attendance (Jankauskiene et al. 2017; Lawson et al. 2017). Furthermore, the majority of treatment required within a three year period post-DGA mirrors this study’s results with restorations taking precedence with a 44.5% requirement, followed by extractions (15%) and endodontic treatment (10%) (Tilja et al. 2019).

Relapse increases the risk of repeat DGAs which have been reported to be as high as 12–37% in north-west England (Goodwin et al. 2015a, b). The six hospitals involved in this study largely provided a direct referral to the DGA without a treatment planning appointment. Other contributory factors proposed for such a high repeat DGA rate include suboptimal attendance within primary care, at hospital dental appointments and DGA appointments as well as a lack of preventative intervention in primary care. Less radical treatment plans under DGA, comprising restorative treatment as opposed to solely exodontia, has also been proposed as a risk factor. One of the hospitals included in this study was a tertiary level hospital which provided treatment for a greater proportion of children with medical and behavioural difficulties unable to access DGA in other facilities (Goodwin et al. 2015a, b). Such medical and behavioural conditions may also preclude access and/or acceptance of dental care within primary care as well as in maintaining good oral hygiene thus increasing the risk of repeat DGAs contributing to the high repeat DGA rate reported.

Conversely, the repeat DGA rate for this study was 0.8% (*n* = 1). Similarly, reports of 1% have been echoed when, as per local procedure, a consultant-led treatment planning appointment is first attended and when dental radiographs are available before or during the DGA (Deery et al. 2015; Lawson et al. 2017). However, the repeat DGA rate in this study may not be reflective of need when suboptimal attendance is considered.

Inclusion criteria for the study included referral by the child’s PCD which would require some form of pre-DGA attendance. However, 10% (*n* = 8) of children pre-DGA were listed as ‘none of the above’ by their PCD suggesting complete lack of attendance which increased to 40% (*n* = 32) post-DGA. The retrospective questionnaire study design relies on the accuracy of dental records and translation of this information to the questionnaire. Therefore, any inaccuracies in reporting or misinterpretation of the questionnaire could partly account for these reported figures. Alternatively, other services may have initiated a DGA referral and the PCD was incorrectly documented as the referrer in the hospital records. Qualitative analysis suggests lack of engagement (*n* = 26), change in primary dental care provider (*n* = 5) and residing abroad (*n* = 1) as factors to help explain post-DGA lack of attendance (40% [*n* = 32]). Nonetheless, this study suggests a significant lack of attendance which will have impacted on the degree of intervention PCDs were able to provide and as such is a general limitation applicable to the results due to the retrospective nature of the study.

One other potential limitation of the study is the low response rate (37.7% [*n* = 43] primary care practices) yielding a small study sample for analysis (26.7% [*n* = 80] children) with relatively fewer children in the 10–16 years age cohort. This uneven distribution amongst age groups is partly reflective of the population with a greater proportion of younger children treatment planned for DGA, as opposed to chairside treatment, due to their young age and associated lack of compliance required for caries management by other modalities.

It is well reported that response rates to questionnaires within dental and medical professions vary greatly with figures reported between 8.5 and 56.8% (Katz et al. 2006; Brøgger et al. 2007; Hardigan et al. 2012; Cook et al. 2016; Turner and Ross 2017). Methods to increase response rate to postal and electronic questionnaires include monetary or non-monetary incentives, a short questionnaire, use of recorded delivery and offer of results (Edwards et al. 2009). The three later strategies were employed in this study but given financial constraints an incentive was not offered but may have increased response rate. Also, the combined postal invitation and online questionnaire may not have suited all invited participants with a solely paper or online format preferred.

Strategies to increase engagement of children with their PCDs could include reorientation of post-DGA advice at pre-GA hospital assessments to increase awareness of the value of post-operative review in primary care in order for children to attend a relatively relaxed setting soon after their DGA. The aim would be to provide a positive experience which may reduce long-standing or DGA-associated anxiety with or without further support for children and family members with anxiety as required. Also, changes to establish a more uniform WNB policy in primary care to support attendance and changes to hospital discharge policy to support high caries risk children through established community oral health programmes, such as the local Childsmile programme, may encourage participation. Finally, positive changes in dental remuneration to reward PCDs for delivering prevention may also support an increase in the incidence of preventative interventions that these high caries risk children could benefit from.

Overall, there are several limitations of this study including: a lower than desired study response rate resulting in a lower study sample for analysis with relatively fewer older children (10–16 years) and a generalised lack of attendance in primary care, especially post-DGA, which appears to be characteristic of this cohort but consequently limiting the degree of intervention PCDs could provide. Further development of future questionnaires to PCDs, such as obtaining confirmation of timely receipt of hospital discharge letters to facilitate arrangement of post-operative review appointments, may help further identify the reason(s) behind such poor attendance post-DGA. Further studies to investigate proposed methods of increasing participation with PCDs, such as liaison with community oral health programmes and/or motivational interviewing, is recommended.

## Conclusion

Considering the limitations of the present study, the following findings from this research highlight improvement is required in strategies employed to support high caries risk children pre- and post-DGA to facilitate a higher incidence of attendance in primary dental care where prevention can be delivered:

- Suboptimal attendance is common within this cohort with engagement declining significantly from pre-DGA to post-DGA and so the null hypothesis is rejected.
- Lack of preventative interventions is common with over one third of children not receiving any form of preventative intervention over 24 months.
- Strategies to support engagement with PCDs could include: liaison with established community oral health programmes, motivational interviewing and changes to NHS dental remuneration.

## Data Availability

Ownership of the data arising from this study resides with the study team. All efforts have been made to ensure that data reported in this study are accurate to the best knowledge of the study team.

## Code availability (software application or custom code)

IBM SPSS Statistics v.24.
